# gpuZoo: Cost-effective estimation of gene regulatory networks using the Graphics Processing Unit

**DOI:** 10.1093/nargab/lqac002

**Published:** 2022-02-08

**Authors:** Marouen Ben Guebila, Daniel C Morgan, Kimberly Glass, Marieke L Kuijjer, Dawn L DeMeo, John Quackenbush

**Affiliations:** Department of Biostatistics, Harvard School of Public Health, Boston, MA, USA; Channing Division of Network Medicine, Department of Medicine, Brigham and Women's Hospital and Harvard Medical School, Boston, MA, USA; Department of Biostatistics, Harvard School of Public Health, Boston, MA, USA; Channing Division of Network Medicine, Department of Medicine, Brigham and Women's Hospital and Harvard Medical School, Boston, MA, USA; Centre for Molecular Medicine Norway (NCMM), Nordic EMBL Partnership, University of Oslo, Oslo, Norway; Department of Pathology, Leiden University Medical Center, Leiden, The Netherlands; Leiden Center for Computational Oncology, Leiden University Medical Center, Leiden, The Netherlands; Channing Division of Network Medicine, Department of Medicine, Brigham and Women's Hospital and Harvard Medical School, Boston, MA, USA; Department of Biostatistics, Harvard School of Public Health, Boston, MA, USA; Channing Division of Network Medicine, Department of Medicine, Brigham and Women's Hospital and Harvard Medical School, Boston, MA, USA

## Abstract

Gene regulatory network inference allows for the modeling of genome-scale regulatory processes that are altered during development, in disease, and in response to perturbations. Our group has developed a collection of tools to model various regulatory processes, including transcriptional (PANDA, SPIDER) and post-transcriptional (PUMA) gene regulation, as well as gene regulation in individual samples (LIONESS). These methods work by postulating a network structure and then optimizing that structure to be consistent with multiple lines of biological evidence through repeated operations on data matrices. Although our methods are widely used, the corresponding computational complexity, and the associated costs and run times, do limit some applications. To improve the cost/time performance of these algorithms, we developed gpuZoo which implements GPU-accelerated calculations, dramatically improving the performance of these algorithms. The runtime of the gpuZoo implementation in MATLAB and Python is up to 61 times faster and 28 times less expensive than multi-core CPU implementation of the same methods. gpuZoo is available in MATLAB through the netZooM package https://github.com/netZoo/netZooM and in Python through the netZooPy package https://github.com/netZoo/netZooPy.

## Graphical Abstract

**Graphical Abstract:**
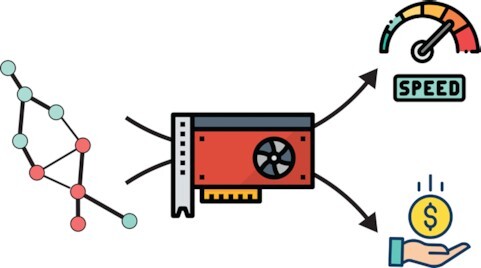
Gene regulatory network (GRN) inference for large studies using PANDA and LIONESS can provide thousands of regulatory network models that can provide insight into biological systems; GPU-optimized code can greatly accelerate the process of network inference and substantially reduce costs.

## INTRODUCTION

Gene regulation defines cell phenotypes and controls cellular functions (1). Transcription factors (TFs) are regulatory proteins that bind to promoter and enhancer regions near a gene and serve to activate or repress the transcriptional process (2,3). A variety of methods have been developed to infer gene regulatory networks from gene expression and other data types (4–7), but PANDA has shown to outperform others in predicting TF binding and identifying phenotype-associated biological processes (8–10). PANDA estimates gene regulatory networks by postulating a TF-gene network based on TF binding sites in the genome and then optimizing that structure to be consistent with TF-TF interaction data and condition-specific gene expression. A number of methods have extended the PANDA framework, including PUMA (11) which includes miRNA regulation (1) by seeding PANDA with miRNA estimated targets, and SPIDER (12) which includes epigenetic data on open chromatin using DNase-seq data. LIONESS (13) allows inference of unique networks for each individual in a population by making iterative calls that assess the effects of excluding individual samples from network reconstruction. These methods have proven quite useful, providing insight into tissue-specific gene regulation (14), explaining sex-specific response to cancer drugs (15), and identifying altered pathways in ovarian cancer (16).

Despite the success of these methods, memory requirements, computational time, and computational cost can present challenges to their use. The algorithms require a large number of matrix operations that were, until recently, reliant on CPUs composed of a relatively small number of computing cores capable of handling only a relatively small number of simultaneous software threads.

Graphics processing units (GPUs) offer an attractive alternative to CPUs, handling repetitive matrix calculations in a faster and more efficient fashion. GPUs have hundreds of cores designed to handle many threads and thus can support the efficient implementation of highly parallel computation in genomics (17) and in network inference (5). To take advantage of this highly efficient, parallelizable, and matrix-optimized hardware, we adapted our methods to run on GPUs; the gpuZoo contains GPU-adapted implementations of PANDA, PUMA, and SPIDER, collectively referred to as gpuPANDA (since all three algorithms share the same core network optimization algorithm) and created gpuLIONESS that implements LIONESS on multi-GPU devices to parallelize the required iterative computation of sample-specific networks. A cost-performance analysis found gpuPANDA to be up to 61 times faster and 28 times less expensive than running multi-threaded CPU implementations of PANDA, with similar performance improvements for gpuLIONESS of about 10x speedup for network modeling on a population of 127 individuals.

## MATERIAL AND METHODS

### The serial implementation of PANDA, PUMA, SPIDER, and LIONESS

PANDA was developed to infer gene regulatory networks by integrating three sources of input data that reflect what we know about the process of transcriptional control, and then iteratively optimizing these to maximize consistency between them. First, because the regulatory process is controlled by transcription factors (TFs) that bind to specific DNA motifs to activate or repress gene expression (3), PANDA takes as input a transcription factor-by-gene ‘regulatory network’ adjacency matrix (*W*_0_) constructed by mapping TF binding sites to a fixed-size window surrounding the transcription start site of each gene. Second is a TF-by-TF ‘cooperativity network’ matrix (*P_0_*), based on ‘protein-protein interaction’ (PPI) data, that reflects the fact that TFs often form multi-protein complexes that carry out regulatory functions (18). The third input is an expression ‘co-regulatory network’ (*C_0_*) consisting of pairwise Pearson Correlation Coefficients (PCC) between genes based on their expression and capturing the fact that genes co-regulated should exhibit similar patterns of expression.

Because the adjacency matrices of the regulatory network (*W*_0_), the cooperativity network (*P_0_*), and the co-regulatory network (*C_0_*) are on different scales, the entries of each are Z-score standardized across both rows and columns. PANDA takes these as input and then iteratively optimizes the consistency between the three input matrices. It first calculates ‘Responsibility’ and ‘Availability’ values for each TF-gene edge and combines these values to update each entry in *W*. Next, it updates the values in *P* and *C*. Each of these updates uses a function based on a modified Tanimoto similarity for continuous variables, which we refer to as the Tfunction; the Tfunction can be conceptualized in terms of large matrix operations, making it amenable to significant improvement using GPU computing (see below and Supplementary Methods).

PANDA (8) computes the final regulatory network (*W_f_*) (Supplementary Figure S1-B) using a step-wise approach defined by a learning rate ($\alpha$) (Supplementary Methods). To better assess performance gains from GPU computing, in addition to the Tfunction we also included seven other commonly used similarity metrics (Euclidean, Squared Euclidean, Standardized Euclidean, City Block, Chebychev, Cosine, and Pearson Correlation) as alternatives for benchmarking purposes (see Supplementary Methods).

PANDA has been extended to incorporate additional regulatory mechanisms. PUMA (11) estimates the regulation of target genes by miRNAs by seeding a modified version of the PANDA algorithm with an estimate of miRNA target predictions in the *W*_0_ network. SPIDER (12) improves the accuracy of PANDA networks by integrating DNase-seq data (which identifies regions of open chromatin) by masking edges in *W*_0_ where chromatin structure indicates TFs are unlikely to bind.

PANDA, PUMA, and SPIDER are aggregate network inference methods that compute a context-specific network by estimating likely regulatory effects across a population. LIONESS (13) builds on these methods and uses linear interpolation to infer individual regulatory networks for each sample in a population. We have used LIONESS with PANDA to infer networks for individuals in large studies, after which the networks are treated as inferred measurements and compared between relevant subgroups (15,19,20).

LIONESS begins by using PANDA to compute an aggregate network (*W*) for all samples using standard PPI (*P_0_*) and motif (*W_0_*) seeds, and a gene co-regulatory matrix *(C_0_*) calculated for all samples; these three seed networks are normalized as part of the standard PANDA preprocessing. Following inference of the initial baseline network, an iterative process is initiated in which LIONESS iteratively leaves out single samples, calculates a model for the population deprived of the *i*^th^ sample (*W^(i)^*), and uses the difference between these two models to estimate the network for the *i*^th^ sample (${W^{( i )}}$) using equation 4 in (13). For each left-out sample *i*, a gene co-regulatory matrix (${C^{( i )}}$) is computed using all samples but sample *i* and normalized. This is then used as input to PANDA (with the same condition-independent motif and PPI prior as previously) to infer a regulatory network for all samples but *i*. The networks with and without sample *i* are used to provide a linear interpolation estimate of the gene regulatory network for the *i*^th^ sample. This process is repeated for all samples in the population, resulting in a collection of network models, one for each sample.

In general, the slowest step in this process is computing and normalizing the co-regulatory network for each of the left-out samples. However, since LIONESS requires computing gene co-expression deprived of sample *i*, we implemented an optimization of the algorithm by computing gene co-expression on-line—inferring the sample-deprived gene co-expression ${C^{( i )}}$ from three initial variables: *m*, a vector representing the mean expression of genes across all samples; *s*, a vector representing the standard deviation in the expression of genes across all samples; and *Cov*, a matrix representing the covariance in expression between pairs of genes across all samples.

First, we use *m* to compute a vector representing the mean expression of genes across all samples except for sample *i*:

(1) $$\begin{equation*}{\rm{\ }}{m^{\left( i \right)}} = \frac{1}{{\left( {n - 1} \right)}}\ \left( {n*m - {G^i}} \right)\end{equation*}$$

where *n* is the number of samples and ${G^i}$ is a vector of the expression of all genes in sample *i*. Next, we use *s* and *m^(i)^* to compute the standard deviation of genes across all samples except for sample *i*:

(2) $$\begin{equation*}{\rm{\ }}{s^{\left( i \right)}} = \sqrt {\left( {{s^2} - \frac{1}{n}*{{\left( {s - {m^{\left( i \right)}}} \right)}^2}*\frac{{n - 1}}{{n - 2}}} \right)} \ \end{equation*}$$

Then we use *Cov* and *m* to compute the covariance across all samples except for sample *i*:

(3) $$\begin{eqnarray*}{\rm{\ }}Co{v^{\left( i \right)}} &=& \frac{1}{{n - 2}}\ *\bigg( {Cov*( {n - 1}) - \ \frac{n}{{n - 1}}*({G^i} - {m^{(i)}}})\nonumber\\ &&*({G^i} - {m^{( i )}})^{\prime}\bigg)\end{eqnarray*}$$

Finally, the sample-deprived co-expression can be computed as follows:

(4) $$\begin{equation*}{\rm{\ }}{C^{\left( i \right)}} = \frac{{Co{v^{\left( i \right)}}}}{{{s^{\left( i \right)}}*{s^{\left( i \right)}}^{\prime}}}\ \end{equation*}$$

Computing the mean, standard deviation, and covariance only once at initiation allows us to infer the co-regulatory network for all subpopulations (each missing a single sample) and avoids having to compute gene co-expression estimates independently for hundreds of samples.

### gpuPANDA and gpuLIONESS

Network inference is implemented in parallel in gpuPANDA and begins by broadcasting data matrices to the GPU device. All subsequent operations required in the network inference step, from measuring the distance between two matrices to element-wise matrix operations such as additions and multiplications (Supplementary Figure S1-A), are performed on the GPU device by distributing them across hundreds of GPU cores using CUDA kernels (21). Each operation is sequentially controlled by the host CPU, which initiates a CUDA kernel through MATLAB/Python GPU interfaces and synchronizes the device at the end of each operation before calling the next operation with the previous result; the resulting network is sent back to the host CPU and stored in memory.

To determine the potential for a regulatory interaction between a TF and gene, PANDA computes a modified Tanimoto similarity *t(x,y)* between the target profile of the TF and co-expressed partners of the target gene, as represented by the networks *W* and *C*. For each TF-gene pair, represented by row *x* of the regulatory adjacency matrix *W* and column *y* of the co-regulatory adjacency matrix *C*, the similarity is computed as follows:

(5) $$\begin{equation*}t\left( {x,y} \right)\ = \frac{{xy^{\prime}}}{{\sqrt {xx^{\prime} + yy^{\prime} - \left| {xy^{\prime}} \right|} }}\ \end{equation*}$$

In gpuPANDA, the similarity between each TF-gene pair is computed in parallel (rather than sequentially as in the original CPU PANDA implementation), meaning that the GPU implementation can theoretically realize a speedup factor on the order of the number of GPU cores (Supplementary Figure S1-A)—although in reality, GPU cores are much slower than CPU cores, and CPU cores can be multithreaded.

The gpuPANDA implementation has additional features designed to optimize GPU memory by considering only half of the symmetrical co-expression matrices. In order to avoid memory transfer overhead, data transfer between host and device are limited to sending input data and getting back the final result (Supplementary Figure S1-C), and in the case of device failure to save intermediary results and restart from the last iteration. In addition, communication between CPU and GPU is initiated for each step of the algorithm to create the CUDA kernels and to synchronize the device before the start of a new operation.

The implementation of gpuLIONESS consists of a series of batch calls to an aggregate network inference approach, such as gpuPANDA, followed by network inference for each sample represented in the dataset. The method takes advantage of the architecture of multi-GPU devices, such as the NVIDIA TESLA K80 and NVIDIA TESLA P100, by assigning the computation of each single-sample network to an individual GPU device in parallel. Both gpuPANDA and gpuLIONESS use the MATLAB GPU interface and Python CuPy library that create CUDA (21) kernels to parallelize computations across GPU cores. To parallelize gpuLIONESS across several non-shared memory units, these libraries embed CUDA kernels in a Message Passing Interface (MPI) process (22) to compute single-sample networks in parallel. This hybrid structure provides two levels of parallelism that ensures message passing of the computed results between non-shared memory processes and within each CUDA process.

gpuZoo which consists of gpuPANDA and gpuLIONESS was implemented in MATLAB (2019a, version 9.6.0, The MathWorks Inc., Natick, MA, USA) as part of the netZooM package (https://github.com/netZoo/netZooM; version 0.5.2) and in Python (version 3.7) as part of the netZooPy package (https://github.com/netZoo/netZooPy; version 0.6.2).

### Benchmarking procedure

The runtime and cost of network generation for the CPU and GPU implementations of PANDA and LIONESS were compared using networks of three sizes: 652 TFs by 1000 genes, 652 TFs by 27,149 genes, and 1,603 TFs by 43,698 transcripts. These roughly correspond to the sizes of a small regulatory network consisting of a subset of protein-coding genes, a network including all human protein-coding genes, and a network including all human transcripts, respectively.

The small size network was derived from the input data used by Lopes-Ramos and colleagues (10) to construct lymphoblast cell line (LCL) regulatory networks using i) expression data from GTEx (23), ii) PPI data from STRINGdb (24), and iii) TF binding predictions derived using FIMO (25) to scan the promoter regions of all gene sequences defined as TSS +/-750bp in the human genome (hg38) for matches to TF PWMs from CIS-BP (3). To create the small network from these data, we restricted the TF binding network to the first 1,000 genes. In the data pre-processing step, we took the intersection of these three input data sources, i.e., the intersection of the TFs in PPI and TF binding motif matrices and the intersection of the genes in the gene co-expression and the TF binding motif matrices; this resulted in *W*, *P*, and *C* matrices that included data for 652 TFs and 1,000 genes.

The protein-coding gene network was also derived from GTEx LCL cell line data, but in the data pre-processing step we used the union of the three complete input data sets rather than restricting to 1,000 genes, which returned the expected 652 TFs while increasing the number of target genes to 27,149 compared to the small network.

Finally, to test the maximal memory capacity of the GPU hardware, we computed a large network consisting of all the known TFs and individual gene transcripts. These individual transcripts reflect the alternative splicing process in which each gene can code for several transcripts. This ‘transcript network’ was based on THP-1 Leukemic monocyte cell line (26), gene expression data from GEO (27) processed in ARCHS4 (28) to obtain transcript levels, a PPI network of 1,603 TFs encoded in the human genome from STRINGdb (24), and the same set of TF binding predictions used in the protein-coding gene network. In the data pre-processing step, we used the union of the three data sources which resulted in a data set consisting of 1,603 TFs and 43,698 transcripts.

In addition to the default Tfunction similarity metric and default learning rate (α = 0.1), we ran PANDA using seven commonly used similarity metrics that can be computed on the GPU (Supplementary Material) and with two additional learning rates (α = 0.2 and α = 0.3). We compared these PANDA runs in terms of computational speed and cost. Our motivation for including additional parameters was twofold. First, we wanted to show that GPU versus CPU results are consistent across different parameters. Second, although we successfully used the similarity metric Tfunction with a learning rate of 0.1 in earlier studies (10,14,15), the cost-effective acceleration provided by gpuPANDA enables the exploration of additional parameter combinations. Therefore, we wanted to ensure that performance gains were guaranteed beyond the standard parameter values.

We assessed the runtime and cost performance of PANDA and gpuPANDA as well as LIONESS and gpuLIONESS, using implementations in MATLAB and Python. To select the optimal machine configuration for the benchmarks, three GPU devices were chosen based on memory size, number of cores, and clock speed. GPU1 (NVIDIA TESLA V100) has 5120 cores and 32 GB of memory, GPU2 (NVIDIA TESLA P100) has 3584 cores and 16 GB of memory, and GPU3 (NVIDIA TESLA K80) has 2496 cores and 12 GB of memory (detailed configurations in Table 1). Testing the implementations on different device sizes allows to find the optimal price-performance configurations. Similarly, CPU instances were selected based on processor clock speed and memory. These instances are categorized in AWS as ‘compute-optimized’ (c5) and ‘memory-optimized’ (r5). We selected a first machine (CPU1) from the ‘compute-optimized’ category with a processor clock speed of 3.6–3.9 Ghz which is the highest in AWS catalog. A second instance (CPU2) was selected for a larger and faster memory access (256 GB as opposed to 96 GB for CPU1). The reason for selecting a compute-optimized and a memory-optimized instance was to determine if significant changes in memory size and processor speed would affect runtime and price-performance. An additional motivation for testing on different machine specifications was to find the optimal configuration for each network model size being tested (small, coding-genes, and transcript network).

**Table 1.** Specification of the hardware units used for benchmarking

Unit	AWS reference	Price ($/hr)	Manufacturer reference	Number of cores	Clock speed (Ghz)	Memory (GB)	Specifications	Region
CPU1	c5d.12xlarge	2.304	2nd generation Intel Xeon Scalable Processors	48	3.6–3.9	96	compute-optimized	us-east-1
CPU2	r5a.8xlarge	1.808	AMD EPYC 7000	32	2.5	256	memory-optimized	us-east-1
GPU1	p3dn.24xlarge	3.902*	Nvidia Tesla V100 Tensor Core	5120	1.53	32	Largest GPU	us-east-1
GPU2	p3.2xlarge	3.06	Nvidia Tesla P100	3584	1.19	16	Large GPU	us-east-1
GPU3	p2.xlarge	0.9	Nvidia Tesla K80	2496	0.52	12	Smaller GPU	us-east-1

In the benchmarks, hyperthreading was enabled to allow all the CPU cores are used to perform computations. EC2 cost corresponds to AWS On-Demand price.

* p3dn.24xlarge has 8 Tesla P100 Tensor Core, the original price of $31.218/hour was divided by 8 to estimate the cost of one unit.

We used a benchmark design that controls for loss of runtime performance due to factors such as co-tenancy and compilation time. Each benchmark was run on a separate virtual machine and without additional processes running concomitantly. In addition, a ‘dry run’ was performed at the beginning of each experiment to allow for the compilation of GPU libraries in MATLAB and Python and to avoid including it in the final runtime.

To reduce the number of comparisons, our approach was to benchmark the transcript model in GPU1 because it could not be loaded in other devices, and to benchmark the coding-genes model on GPU1 and GPU2 for the same reasons. Finally, we benchmarked the small model on GPU2 and GPU3 because with larger devices, the initialization time could exceed the computation time.

All analyses were performed on Amazon Web Services (AWS) (accessed on 06/2021) on Python (version 3.7), and MATLAB (version 2019a) in Ubuntu 18.04 and Windows 10 that enables MATLAB memory benchmarking. The cost was computed as the price of AWS instance multiplied by the runtime in seconds. We chose to benchmark the tools using AWS cloud computing because it offers a wide variety of General-Purpose GPUs and provides tools for estimating computing cost.

## RESULTS

We first ran the MATLAB implementations of PANDA and gpuPANDA on our three test networks using three learning rate values (α = 0.1, α = 0.2, α = 0.3) with calculations in single and double precision; we also ran these methods with each of the eight similarity metrics.

**Figure 1.**
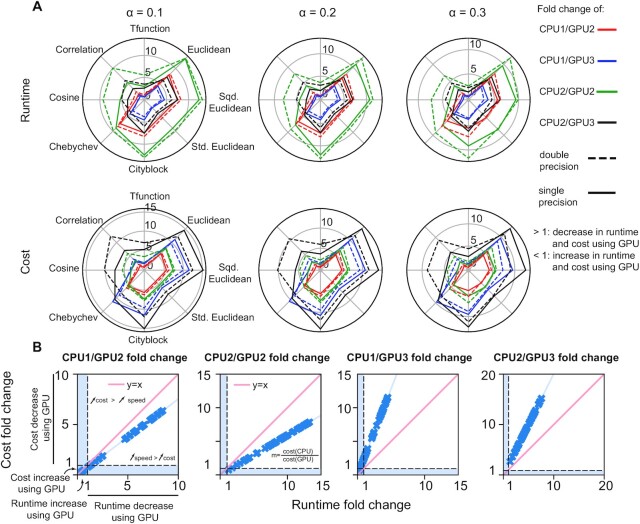
Runtime and cost performance of gpuPANDA in the small network. **(A**). Runtime (first row) and cost (second row) fold change between CPU1, CPU2, GPU2, and GPU3. The results are reported as CPU/GPU fold change, therefore, a fold change larger than 1 indicates a decrease of cost or runtime using GPU. Conversely, a fold change less than 1 indicates an increase in cost or runtime using GPU. (**B**). Rate of cost fold change as an effect of runtime fold change in the small network in single and double precision. The blue area represents the case when CPU/GPU fold change is less than 1 indicating an increase in cost and/or runtime of GPU computation over CPU.

For the small network that includes 652 TFs and 1,000 genes, both PANDA on CPU1 and CPU2 and gpuPANDA running on the GPU2 and GPU3 devices were able to infer gene regulatory network models that were identical to one another as determined by the absolute value of the largest difference (Supplementary Figure S2). This was true using all eight similarity metrics and running in both single and double precision. gpuPANDA demonstrated significant advantages in both runtime (up to 7 times faster with GPU3 in comparison to CPU2; Supplementary Tables S1 and S2) and cost (up to 15 times less expensive with GPU3 in comparison to CPU2; Figure 1-A, Supplementary Tables S3 and S4). To further determine the effect of scaling on runtime and cost, we compared the rate of speedup to the rate of cost decrease. In comparison to PANDA on CPU1 and CPU2, the speedup of gpuPANDA using GPU2 outpaced the decrease in cost (Figure 1-B) such that a two-fold speedup comes with < two-fold decrease in cost. However, comparing PANDA on CPU1 and CPU2 to gpuPANDA on GPU3 showed a decrease in cost at a larger rate than speedup, which means that achieving a two-fold speedup with GPU comes with > two-fold decrease in cost. The reason for this is that small networks do not require large devices, and can be efficiently computed with the smaller and less expensive GPU3.

**Figure 2.**
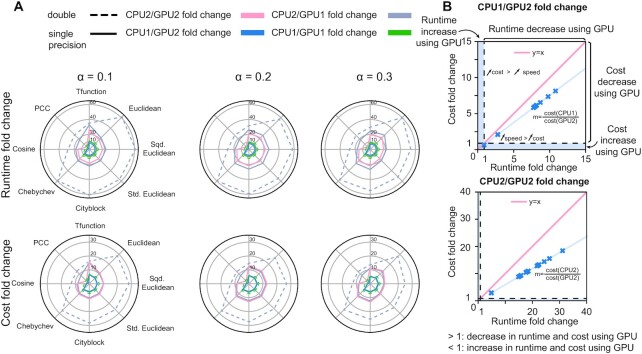
Runtime and cost performance of GPUs and CPUs on the protein coding-genes model. (**A**). Fold change of runtime as a function of cost between CPU1 and GPU2 and CPU2 and GPU2 in single precision and for three values of learning rate (α). A CPU/GPU fold change larger than 1 indicates a decrease in cost or runtime using GPU. Fold change smaller than 1 indicates an increase in runtime or cost using GPU. (**B**). Effect of runtime fold change on cost fold change between CPU1 and GPU2 (top panel) and CPU2 and GPU2 (bottom panel). The blue area indicates the cases where using GPU is slower and/or more expensive than CPU (fold change < 1).

For the network modeled on protein-coding genes, GPU acceleration was possible in GPU1 but only in single precision with GPU2 due to memory limitations. gpuPANDA had an up to nine-fold decrease in runtime and a seven-fold decrease in cost when comparing GPU2 and the compute-optimized device CPU1 (Figure 2-A). For the memory-optimized CPU2, the speedup reached up to 26-fold with a decrease in cost of up to 15-fold (Figure 2-A). This was particularly clear with the modified Tanimoto (Tfunction) similarity metric at the default learning rate of 0.1. An analysis of cost fold decrease rates as a function of speedup rates (Figure 2-B) showed that CPU1/GPU2 and CPU2/GPU2 had a runtime decrease at a faster rate than cost decrease. Similarly, GPU1 was up to 12 times faster than CPU1 and up 61 times faster than CPU2 particularly in double precision computation using the Euclidean distance, which corresponded to a seven-fold and 28-fold reduction in cost, respectively.

**Figure 3.**
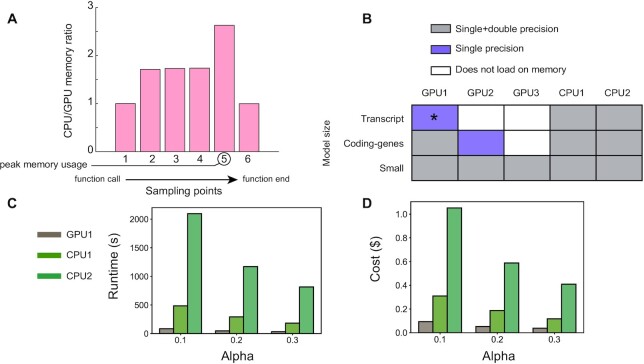
GPU performance on transcript model and memory benchmark. (**A**). Memory usage of GPU implementation in comparison to CPU implementation. (**B**). Tested network precision for the tested hardware using the small network, protein-coding genes network, and transcript regulatory gene network. (**C**). Runtime and D-cost of running transcript model on GPU1, CPU1, and CPU2 in single precision. Average coefficient of variation across learning rates in runtime is 0.4% for CPU1, 0.5% for CPU2, and 0.2% for CPU3. *Single precision computation on GPU1 converges with Tfunction only.

We designed the GPU code to optimize memory usage. Specifically, we measured the memory requirements of PANDA and gpuPANDA across six sampling points after the function call (Figure 3-A) and found a 2.6-fold decrease in memory usage with our optimized GPU code. However, despite this improvement, neither the GPU2 and GPU3 configuration had sufficient memory to load the input matrices of the transcript network and perform operations using either single or double precision (Figure 3-B). In addition, we could load and compute the network on GPU1 in single precision only for the Tfunction similarity metric. Benchmarking against CPU1 and CPU2 in this setting revealed a 24-fold decrease in runtime (Figure 3-C) and 11-fold decrease in cost (Figure 3-D). Finally, to test the reproducibility of these results, we repeated the CPU and GPU runs three times using CPU1 and GPU1 as representatives of each category. CPU1 had an average coefficient of variation (CV) of 0.31% and GPU1 had an average CV of 0.37% across experiments. (Supplementary Figure S7).

In addition to testing the MATLAB code, we tested the Python implementations of PANDA and gpuPANDA on the small network. We found similar results to those described above that were based on the MATLAB implementation. For example, calculating single precision networks using PANDA on CPU2 and gpuPANDA on GPU3, gpuPANDA was more than 10 times faster than the CPU implementation. We also found the output networks to be identical, with the largest absolute difference in edge weights equal to 3.5 × 10^–5^ (Supplementary Figure S3).

Finally, we tested LIONESS and gpuLIONESS in MATLAB. Both LIONESS and gpuLIONESS perform a series of batch calls, in our case to PANDA and gpuPANDA, respectively. In estimating 127 single precision individual sample networks based on the small network dataset, we found gpuLIONESS to be 10.3 times faster on GPU2 compared to LIONESS on CPU2 and 3.6 times faster than CPU1. On GPU1, gpuLIONESS was 24.9 times faster than CPU2 and 8.7 times faster than CPU1 (Supplementary Figure S4-A). The largest absolute difference between the CPU and GPU network edge weights was 0.015, which was less than 0.01% of that edge weight, while the average absolute difference was 6.5 × 10^–5^ (Supplementary Figure S4-B). We also combined GPU speedup with an additional algorithmic improvement consisting of deriving the co-expression network on-line (Eq1-Eq4), i.e., without having to recompute it for every sample (Supplementary Figure S4), although this approach did not lend reduced runtime. Finally, we also tested a multi-GPU implementation that distributes single-sample network inference across several GPU devices. Benchmarks on an AWS p3.8xlarge instance with four GPUs revealed a linear speedup as a function of the number of devices (Table 2).

**Table 2.** Runtime of gpuLIONESS in a multi-GPU setting

Number of GPUs	Runtime (s)
1	12,307
2	6,441
4	3,392

The coding-genes network was benchmarked in a multi-GPU setting using a learning rate of 0.1 and in single precision to generate 127 single-sample networks using a p3.8xlarge AWS instance (4 Tesla V100 GPUs, us-east-1, accessed on 10/2021).

## DISCUSSION

As the sample sizes for genomic and multi-omic data studies grow, we have the opportunity to develop increasingly accurate models of the potential causes of various diseases and phenotypic traits. However, the computational complexity, time, and cost of building such models has become a limiting factor in many applications. The development of PANDA, PUMA, SPIDER, and LIONESS as techniques for inferring accurate regulatory models has allowed the exploration of gene regulation in health and disease. However, the use of these models has been limited by the availability of computational resources. For example, using PANDA and LIONESS to generate more than 9,435 individual sample networks (19) using data from GTEx v6 initially took multiple months running on a conventional multi-CPU cluster; rerunning those networks in response to a question from referees took more than six weeks (after having optimized the CPU code). Our interest in repeating this analysis with GTEx v8 and with other large datasets, underscores the need for additional computational improvements.

Although PANDA, PUMA, and SPIDER are not massively parallel algorithms as the estimation of edges in the final network cannot be structured into independent tasks, they include a series of operations such as matrix multiplication and distance computation that make them amenable to GPU acceleration (9). gpuPANDA represents an adaptation of these methods that parallelizes the large matrix operations in each iteration of the network inference and refinement process. The implementation of gpuLIONESS extends this further by distributing the calculation of PANDA networks for each leave-one-out data subset across the available GPU devices, such that the computation of each individual sample network is distributed across the cores available within each device.

Improving runtime was a major motivation for creating GPU implementations of PANDA and LIONESS. By taking advantage of Python and MATLAB GPU interfaces to CUDA (21), gpuPANDA reduced memory use by 2.6-fold relative to the CPU implementation, in part because it is able to take advantage of symmetries in the co-expression network (which is generally the largest network). We recognize that we might be able to further reduce memory usage by sending intermediate results from device to host to free space for the next iteration. However, we chose not to do so because the associated I/O would considerably increase computation time and, consequently, cost. Furthermore, gpuZoo implementations use MATLAB GPU interface and Python's CuPy library to create CUDA kernels for each parallel operation. Although a pure CUDA implementation would offer better memory usage and runtime performance, using GPU libraries still achieves significant improvements while providing great ease-of-use for the users of these tools to run existing pipelines by simply setting the ‘compute’ argument to ‘gpu.’ These libraries also enable several features such as the distribution across several nodes (AWS EC2 instances or cluster nodes) without additional requirements on the user end. Therefore, using integrated GPU libraries offers an optimal trade-off between accessibility of these tools and performance.

Despite these improvements, neither of the GPU devices, including the larger memory GPU1 (32 GB), was able to load the data for the largest transcript network in double precision (43,698 transcripts and 1,603 TFs, Figure 3-D). This is not surprising, given the size of the co-expression network. This network requires 15.2 GB of memory (43,698*43,698*8 bytes) and performing additional operations on it and on other input data requires holding temporary variables of the same size, such as the ones required during matrix normalization. However, this should not pose a major barrier to the use of gpuZoo since most network inference modeling only include the 20,000–30,000 protein-coding genes. Additionally, most pipelines would further eliminate genes not expressed in a particular tissue during data preprocessing. For example, the majority of our earlier investigations (14,15,19) fall within the size of the protein-coding genes network, for which the computations carried with the modified Tanimoto similarity (Tfunction) had the largest speedup with gpuPANDA. With GPU3, gpuZoo was not able to load the protein-coding genes network (652 TFs and 27,149 genes), and with GPU2 it was only able to load it in single precision. However, the loss of double precision in the matrix calculations does not produce major changes in the overall network estimation and likely has a much less significant effect than noise in measurements of gene expression (Supplementary Figure S5).

Computing 127 single-precision, sample-specific networks using gpuLIONESS for the protein-coding genes network on GPU2 was 10.3 times faster than CPU2 and 3.6 faster than CPU1. The reason for a lower performance in gpuLIONESS compared to gpuPANDA is due to an additional memory-consuming step. When inferring a sample-specific network using PANDA together with LIONESS, there is an additional step that requires recomputing and normalizing the gene co-expression network for each sample, which requires large memory resources due to the size of the co-expression network. Since GPU2 has limited memory, this step was performed in CPU and the resulting data is sent to GPU in each iteration. Using the extra memory of GPU1, this step could be performed on device, which improved the speedup to 24.9-fold in comparison to CPU2 and 8.7-fold in comparison to CPU1. Furthermore, we tested the distribution of single-sample LIONESS network inference using several GPU devices. Using the multi-GPU approach, we found that runtime scales linearly with the number of devices because gpuLIONESS is massively parallel and no additional communication overhead was expected. Since the price of multi-GPU instances is scaled in the same way (p3.2xlarge, 1 GPU, $3.06/hour; p3.8xlarge, 4GPUs, $12.24/hour), the cost-performance advantages are maintained in a multi-GPU setting.

We also investigated combining GPU acceleration with computing gene co-expression on-line. We did not see an improvement in the total runtime in our tested networks. However, we have investigated whether this approach could be beneficial when the number of samples increases relative to the number of genes. Running a comparison between co-expression and on-line co-expression on a 1,000-variable random network showed similar performance (Supplementary Figure S6) when the number of samples was 0.5% the number of genes, which is about the ratio used in our study (127 samples and 27,149 genes). However, increasing the sample-to-genes ratio yielded a 2.45 speedup when the number of samples was equal to number of genes, with acceleration starting at 50% (1.5 speedup). We recently computed sample-specific gene regulatory networks using data from the Connectivity Map across 170,013 experiments on 12,328 genes in two days by combining acceleration from GPU and on-line co-expression (29), which would have required several weeks using CPU.

Finally, to test the accuracy of gpuLIONESS, we found that the largest absolute difference between the edge weights of single-sample gpuLIONESS networks was 0.015 which is larger than the difference between PANDA and gpuPANDA networks in single precision (∼1 × 10^–5^), but the average error was equal to 6.5 × 10^–5^, which is within the order of single precision computation. For the small network, despite a greater increase in network inference speed with GPU2, the smaller GPU3 was more cost-effective for a similar performance (Figure 1-A, Supplementary Tables S1 and S3). In particular, computing gene regulatory networks using the similarity metric Tfunction on GPU2 was less efficient than GPU3 and CPU1, because initializing a large device requires more time than the computation itself.

Comparing inference of regulatory networks using gpuPANDA on three GPU architectures and PANDA on two CPUs, each with different specifications, allowed us to understand the effects of processor clock speed and memory size and access speed on the final runtime and cost. In particular, the CPU machines on which we ran PANDA were significantly different: CPU1, the compute-optimized machine has a faster processing speed and 96 GB of RAM, while the memory-optimized CPU2 has slower CPUs but far greater and faster accessible memory (256 GB) (Table 1). However, performance can be enhanced by taking advantage of the large offering of instances in the AWS catalog which allows users to further fine tune machine configurations that deliver optimal price-performance.

The main drawback of these implementations is that they are unable to process networks with more than 20,000 genes in double precision. However, we found that the differences between single precision and double precision networks remain within the order of single precision, which indicates that neither hardware specifications nor the software implementation account for additional deviation in precision than what is expected (Supplementary Figure S5). Therefore, computing in single precision when GPU memory is limited could be a viable approach for networks that cover more than protein-coding genes. These results could even support the use of half-precision (2 bytes) in memory-scarce settings, but additional experiments have to be done to ensure the accuracy of computational and biological findings.

Overall, we found that gpuZoo offers an optimal cost-performance solution for the estimation of batches of gene regulatory networks. These implementations allow the inference of gene regulatory networks in large-scale genomic studies such as TCGA (30), the Connectivity Map (31), and the GTEx project (23). Moreover, the rapid pace of improvement of GPU devices (32) such as the NVIDIA A100 (40 GB of memory), available through p4d AWS instances, will soon enable cost-effective, large-scale network inference in double precision. Finally, gpuZoo tools enable biological discovery by providing a computational engine that supports our recent endeavor to reconstruct gene regulatory networks across human conditions (29) such as cancer human tissues and cell lines (https://grand.networkmedicine.org).

## DATA AVAILABILITY

gpuZoo (gpuPANDA, gpuPUMA, gpuSPIDER, and gpuLIONESS) is available through the Network Zoo package (netZoo; netzoo.github.io) in MATLAB (netZooM v0.5.2) at https://github.com/netZoo/netZooM with a step-by-step tutorial https://github.com/netZoo/netZooM/tree/master/tutorials, and in Python (netZooPy v0.6.2) https://github.com/netZoo/netZooPy with a tutorial https://github.com/netZoo/netZooPy/tree/master/tutorials.

The code of the benchmarks is available at https://github.com/QuackenbushLab/gpuzoo, and the corresponding data is available at https://netzoo.github.io/zooanimals/gpuzoo/.

## Supplementary Material

lqac002_Supplemental_Files

## References

[1] Hobert O. Gene regulation by transcription factors and microRNAs. Science. 2008; 319:1785–1786. 18369135 10.1126/science.1151651

[2] Zeitlinger J. Seven myths of how transcription factors read the cis-regulatory code. Current Opinion in Systems Biology. 2020; 23:22–31. 33134611 10.1016/j.coisb.2020.08.002 PMC7592701

[3] Lambert S.A. , Jolma A., Campitelli L.F., Das P.K., Yin Y., Albu M., Chen X., Taipale J., Hughes T.R., Weirauch M.T. The human transcription factors. Cell. 2018; 175:598–599. 30290144 10.1016/j.cell.2018.09.045

[4] Irrthum A. , Wehenkel L., Geurts P. Inferring regulatory networks from expression data using tree-based methods. PLoS One. 2010; 5:e12776. 20927193 10.1371/journal.pone.0012776 PMC2946910

[5] He J. , Zhou Z., Reed M., Califano A. Accelerated parallel algorithm for gene network reverse engineering. BMC Syst. Biol. 2017; 11:85–97. 28950860 10.1186/s12918-017-0458-5 PMC5615246

[6] Haury A.-C. , Mordelet F., Vera-Licona P., Vert J.-P. TIGRESS: trustful inference of gene regulation using stability selection. BMC Syst. Biol. 2012; 6:1–17. 23173819 10.1186/1752-0509-6-145 PMC3598250

[7] Ruyssinck J. , Geurts P., Dhaene T., Demeester P., Saeys Y. NIMEFI: gene regulatory network inference using multiple ensemble feature importance algorithms. PLoS One. 2014; 9:e92709. 24667482 10.1371/journal.pone.0092709 PMC3965471

[8] Glass K. , Huttenhower C., Quackenbush J., Yuan G.C. Passing messages between biological networks to refine predicted interactions. PLoS One. 2013; 8:e64832. 23741402 10.1371/journal.pone.0064832 PMC3669401

[9] Glass K. , Quackenbush J., Kepner J. 2015 IEEE High Performance Extreme Computing Conference (HPEC). 2015; IEEE 1–6.

[10] Lopes-Ramos C.M. , Paulson J.N., Chen C.Y., Kuijjer M.L., Fagny M., Platig J., Sonawane A.R., DeMeo D.L., Quackenbush J., Glass K. Regulatory network changes between cell lines and their tissues of origin. BMC Genomics. 2017; 18:723. 28899340 10.1186/s12864-017-4111-x PMC5596945

[11] Kuijjer M.L. , Fagny M., Marin A., Quackenbush J., Glass K. PUMA: PANDA using MicroRNA associations. Bioinformatics. 2020; 36:4765–4773. 32860050 10.1093/bioinformatics/btaa571 PMC7750953

[12] Sonawane A.R. , DeMeo D.L., Quackenbush J., Glass K. Constructing gene regulatory networks using epigenetic data. npj Syst Biol Appl. 2020; 7:45. 10.1038/s41540-021-00208-3 PMC8660777 34887443

[13] Kuijjer M.L. , Tung M.G., Yuan G., Quackenbush J., Glass K. Estimating sample-specific regulatory networks. iScience. 2019; 14:226–240. 30981959 10.1016/j.isci.2019.03.021 PMC6463816

[14] Sonawane A.R. , Platig J., Fagny M., Chen C.Y., Paulson J.N., Lopes-Ramos C.M., DeMeo D.L., Quackenbush J., Glass K., Kuijjer M.L. Understanding tissue-specific gene regulation. Cell Rep. 2017; 21:1077–1088. 29069589 10.1016/j.celrep.2017.10.001 PMC5828531

[15] Lopes-Ramos C.M. , Kuijjer M.L., Ogino S., Fuchs C.S., DeMeo D.L., Glass K., Quackenbush J. Gene regulatory network analysis identifies sex-linked differences in colon cancer drug metabolism. Cancer Res. 2018; 78:5538–5547. 30275053 10.1158/0008-5472.CAN-18-0454 PMC6169995

[16] Glass K. , Quackenbush J., Spentzos D., Haibe-Kains B., Yuan G.C. A network model for angiogenesis in ovarian cancer. BMC Bioinf. 2015; 16:115. 10.1186/s12859-015-0551-y PMC4408593 25888305

[17] Taylor-Weiner A. , Aguet F., Haradhvala N.J., Gosai S., Anand S., Kim J., Ardlie K., Van Allen E.M., Getz G. Scaling computational genomics to millions of individuals with GPUs. Genome Biol. 2019; 20:1–5. 31675989 10.1186/s13059-019-1836-7 PMC6823959

[18] Zhang Y. , Song C., Zhang Y., Wang Y., Feng C., Chen J., Wei L., Pan Q., Shang D., Zhu Y. TcoFBase: a comprehensive database for decoding the regulatory transcription co-factors in human and mouse. Nucleic Acids Res. 2021; 50:D391–D401. 10.1093/nar/gkab950 PMC8728270 34718747

[19] Lopes-Ramos C.M. , Chen C.Y., Kuijjer M.L., Paulson J.N., Sonawane A.R., Fagny M., Platig J., Glass K., Quackenbush J., DeMeo D.L. Sex differences in gene expression and regulatory networks across 29 human tissues. Cell Rep. 2020; 31:107795. 32579922 10.1016/j.celrep.2020.107795 PMC7898458

[20] Lopes-Ramos C.M. , Belova T., Brunner T., Ben Guebila M., Osorio D., Quackenbush J., Kuijjer M.L. Regulation of PD1 signaling is associated with prognosis in glioblastoma multiforme. Cancer Res. 2021; 81:5401–5412. 34493595 10.1158/0008-5472.CAN-21-0730 PMC8563450

[21] Nickolls J. , Buck I., Garland M., Skadron K. Scalable parallel programming with CUDA. Queue. 2008; 6:40–53.

[22] Message Passing Interface Forum MPI: a message-passing interface standard. 1994; Knoxville,TN University of Tennessee www.netlib.org/utk/people/JackDongarra/PAPERS/059_1994_mpi-a-message-passing-interface-standard.pdf.

[23] GTEx Consortium, Laboratory, Data Analysis &Coordinating Center (LDACC)—Analysis Working Group, Statistical Methods groups—Analysis Working Group, Enhancing GTEx (eGTEx) groups, NIH Common Fund, NIH/NCI, NIH/NHGRI, NIH/NIMH, NIH/NIDA, Biospecimen Collection Source Site—NDRI Genetic effects on gene expression across human tissues. Nature. 2017; 550:204–213. 29022597 10.1038/nature24277 PMC5776756

[24] Szklarczyk D. , Gable A.L., Lyon D., Junge A., Wyder S., Huerta-Cepas J., Simonovic M., Doncheva N.T., Morris J.H., Bork P. et al. STRING v11: protein-protein association networks with increased coverage, supporting functional discovery in genome-wide experimental datasets. Nucleic Acids Res. 2019; 47:D607–D613. 30476243 10.1093/nar/gky1131 PMC6323986

[25] Grant C.E. , Bailey T.L., Noble W.S. FIMO: scanning for occurrences of a given motif. Bioinformatics. 2011; 27:1017–1018. 21330290 10.1093/bioinformatics/btr064 PMC3065696

[26] Bosshart H. , Heinzelmann M. THP-1 cells as a model for human monocytes. Ann. Transl. Med. 2016; 4:10.21037/atm.2016.08.53 PMC5124613 27942529

[27] Edgar R. , Domrachev M., Lash A.E. Gene expression omnibus: NCBI gene expression and hybridization array data repository. Nucleic Acids Res. 2002; 30:207–210. 11752295 10.1093/nar/30.1.207 PMC99122

[28] Lachmann A. , Torre D., Keenan A.B., Jagodnik K.M., Lee H.J., Wang L., Silverstein M.C., Ma’ayan A. Massive mining of publicly available RNA-seq data from human and mouse. Nat. Commun. 2018; 9:1–10. 29636450 10.1038/s41467-018-03751-6 PMC5893633

[29] Ben Guebila M. , Lopes-Ramos C.M., Weighill D., Sonawane A.R., Burkholz R., Shamsaei B., Platig J., Glass K., Kuijjer Marieke L., Quackenbush J. GRAND: a database of gene regulatory network models across human conditions. Nucleic Acids Res. 2022; 50:D610–D621. 34508353 10.1093/nar/gkab778 PMC8728257

[30] Weinstein J.N. , Collisson E.A., Mills G.B., Shaw K.R.M., Ozenberger B.A., Ellrott K., Shmulevich I., Sander C., Stuart J.M. The cancer genome atlas pan-cancer analysis project. Nat. Genet. 2013; 45:1113–1120. 24071849 10.1038/ng.2764 PMC3919969

[31] Subramanian A. , Narayan R., Corsello S.M., Peck D.D., Natoli T.E., Lu X., Gould J., Davis J.F., Tubelli A.A., Asiedu J.K. et al. A next generation connectivity map: L1000 platform and the first 1,000,000 profiles. Cell. 2017; 171:1437–1452. 29195078 10.1016/j.cell.2017.10.049 PMC5990023

[32] Bridges R.A. , Imam N., Mintz T.M. Understanding GPU power: a survey of profiling, modeling, and simulation methods. ACM Comput. Surv. 2016; 49:41.

